# Does placental invasiveness lead to higher rates of malignant transformation in mammals? Response to: ‘Available data suggests positive relationship between placental invasion an malignancy’

**DOI:** 10.1093/emph/eoaa025

**Published:** 2020-07-17

**Authors:** Amy M Boddy, Lisa M Abegglen, Athena Aktipis, Joshua D Schiffman, Carlo C Maley, Carmel Witte

**Affiliations:** Department of Anthropology, University of California, Santa Barbara, Santa Barbara, CA, USA; Department of Pediatrics and Huntsman Cancer Institute, University of Utah, Salt Lake City, UT, USA; Department of Psychology, Arizona State University, Tempe, AZ, USA; Arizona Cancer and Evolution Center, Biodesign Institute, Arizona State University, Tempe, AZ, USA; Department of Pediatrics and Huntsman Cancer Institute, University of Utah, Salt Lake City, UT, USA; Arizona Cancer and Evolution Center, Biodesign Institute, Arizona State University, Tempe, AZ, USA; Disease Investigations, San Diego Zoo Global, San Diego, CA, USA

In our study, *Lifetime cancer prevalence and life history traits in mammals*, we reported the prevalence of neoplasia and malignancy in a select group of mammals housed at San Diego Zoo Global from 1964 to 1978 and 1987 to 2015 [1]. We also used these data to evaluate associations between life history traits and measures of population health. Our analysis showed placental invasiveness could not predict the proportion of animals diagnosed with neoplasia or malignancy. In a response to our article, Drs Wagner and colleagues describe a different calculation to test for a relationship between placental invasiveness and malignancy. They identified and included previously published veterinary neoplasia and malignancy data with our published dataset and suggest a positive relationship between placental invasiveness and development of malignancy (referred to as malignancy rate in Wagner and colleagues’ response). These data provided support for the Evolved Levels of Invasiveness (ELI) hypothesis [2]. We are pleased that other investigators find our data useful, and wholeheartedly agree with Drs Wagner and colleagues in the need to identify more data on cancer in a wide variety of species. Notwithstanding, this updated analysis brings up a number of topics that we would like to address. 

According to the Evolved Levels of Invasiveness hypothesis [2], Drs Wagner and colleagues propose an association between rate of malignancy development and placentation within a subset of animals already diagnosed with neoplasia. While we agree this is an interesting question that warrants further investigation, this was not the original question that our study was designed to answer. Our approach calculated malignancy prevalence among necropsied animals, as a measure of disease burden and population health. Indeed, these two questions (and the methods for calculating these questions) complement each other and can improve our understanding of malignancy in mammals. Drs Wagner and colleagues draw an important distinction between the development of a neoplasia and the transition from a benign neoplasm to a malignant neoplasm, called ‘transformation’ in cancer biology. With their calculation, they test if placental invasiveness predicts malignant transformation in mammals. We agree with the importance of this transition in neoplastic progression, and given the definition of malignancy as an invasive and/or metastatic neoplasm, it seems reasonable that transformation might be associated with the biology of placental invasion. Additionally, it should be clarified that not all malignant tumors are metastatic (i.e. travel to distant organs) and there may be different mechanisms underlying development to metastasis, as well.

Combining the results of the two approaches outlined above, the current data suggest placental type does not explain the differences in neoplasia or malignancy prevalence in mammals. However, of the animals that develop benign tumors, species with the more invasive placenta (hemochorial) may have higher rates of malignant transformation. In addition, the processes causing benign tumors to become malignant vary for different cancers subtypes. For example, previous data suggest the malignancy transformation in non-glandular epithelial cancer is high (>83%) for all placental types, but rates of transformation between species (cow/horse vs dog/cat) from benign to malignant diverge for skin and glandular epithelial cancers [3, 4]. These results suggest cellular and tissue origin of the tumor may be an important component in the link between the risk of malignancy transformation and placental invasiveness.

We would like to note a caveat about the data used in Drs Wagner and colleagues’ responses. In the re-analysis, the authors added malignancy and neoplasia data from four additional species (cat, dog, horse and cow) from 12 United States and Canadian Colleges of Veterinary Medicine, collected from 1964 to 1969 and published in by Priester and Mantel [4] in 1971. We suggest caution when using data from domesticated species, as the artificial selective pressures of domestication may have overwhelmed the natural selective pressures of cancer. It also appears that the data added in Wagner and colleagues’ re-analyses were restricted to cancers of the skin, glandular epithelium, non-glandular epithelium and connective tissues (e.g. 208 malignant tumors of bovines [3] compared to the 401 reported by Priester and Mantel [4]). Our cancer data were not restricted by anatomical or tissue type. Drs Wagner and colleagues concluded the current dataset is too small to explicitly test their predictions. A larger dataset, including high-quality data from managed populations, analyzed in collaboration with board-certified veterinary pathologists, will help move comparative oncology and the evolution of malignancy forward.

**Conflict of interest:** Dr. Schiffman is co-founder, shareholder, and employed by PEEL Therapeutics, Inc., a company developing evolution-inspired medicines based on cancer resistance in elephants. Dr. Abegglen is share-holder and consultant to PEEL Therapeutics, Inc.
